# Ventilatory Response at Rest and During Maximal Exercise Testing in Patients with Severe Obesity Before and After Sleeve Gastrectomy

**DOI:** 10.1007/s11695-020-04944-z

**Published:** 2020-08-26

**Authors:** Nicola Borasio, Daniel Neunhaeuserer, Andrea Gasperetti, Claudia Favero, Veronica Baioccato, Marco Bergamin, Luca Busetto, Mirto Foletto, Roberto Vettor, Andrea Ermolao

**Affiliations:** 1 Sports and Exercise Medicine Division, Department of Medicine, University of Padova, Via Giustiniani 2, 35128 Padova, Italy; 2 Department of Medicine, Internal Medicine 3, University of Padova, Via Giustiniani 2, 35128 Padova, Italy; 3 Week Surgery, Bariatric Unit, University of Padova, Via Giustiniani 2, 35128 Padova, Italy

**Keywords:** Bariatric surgery, Cardiopulmonary exercise test, Spirometry, Lung function, Ventilation

## Abstract

**Introduction:**

Sleeve gastrectomy (SG) has become a widespread treatment option in patients affected by severe obesity. However, studies investigating the impact of the subsequent weight loss on the ventilatory response at rest and during physical exercise are lacking.

**Methods:**

This is an observational study on 46 patients with severe obesity (76% females), comparing parameters of ventilatory function 1 month before and 6 months after SG. Patients were first evaluated by resting spirometry and subsequently with an incremental, maximal cardiopulmonary exercise test (CPET) on treadmill.

**Results:**

The important weight loss of 26.35 ± 6.17% of body weight (BMI from 43.59 ± 5.30 to 32.27 ± 4.84 kg/m^2^) after SG was associated with a significant improvement in lung volumes and flows during forced expiration at rest, while resting ventilation and tidal volume were reduced (all *p* ≤ 0.001). CPET revealed decreased ventilation during incremental exercise (*p* < 0.001), with a less shallow ventilatory pattern shown by a lower increase of breathing frequency (∆BF_rest to AT_ *p* = 0.028) and a larger response of tidal volume (∆TV_AT to Peak_ *p* < 0.001). Furthermore, a concomitant improvement of the calculated dead space ventilation, VE/VCO_2_ slope and peripheral oxygen saturation was shown (all *p* ≤ 0.002). Additionally, the increased breathing reserve at peak exercise was associated with a lower absolute oxygen consumption but improved exercise capacity and tolerance (all *p* < 0.001).

**Conclusion:**

The weight loss induced by SG led to less burdensome restrictive limitations of the respiratory system and to a reduction of ventilation at rest and during exercise, possibly explained by an increased ventilatory efficiency and a decrease in oxygen demands.

## Introduction

The worldwide increasing prevalence of obesity represents a major health burden and is associated with an increased risk for many cardiovascular and metabolic diseases and comorbidities [1]. Adipose tissue accumulation on chest wall, abdomen and in proximity of the upper airways may have an impact on lung function, even in the absence of a specific pulmonary disease. This may cause a limitation in thorax and diaphragm mobility, required for appropriate chest wall compliance and ventilatory efficiency [2, 3]. Indeed, impaired lung volume, expiratory flow, airway resistance and functional residual capacity were previously reported following even a modest weight increase [2, 4, 5]. It is worth mentioning that an associated marked reduction in expiratory reserve volume may lead to an increased alveolar surface tension and small areas of atelectasis resulting in reduced lung compliance and a potential ventilation-perfusion mismatch [2]. The causes of decreased dynamic lung volumes could be both mechanical and inflammatory [2, 4]. Indeed, obesity is correlated with increased levels of pro-inflammatory adipokines, which also affect lower airways, by promoting inflammatory processes, including local oedema [4, 6, 7]. The associated alterations in pulmonary flows and the increased respiratory muscle workload affect ventilation during exercise as well. Thus, patients with obesity tend to dynamically hyperinflate during exercise to counteract significant expiratory flow limitation, transferring the tidal volume (TV) to a more compliant portion of the respiratory system. However, hyperinflation reduces the efficiency of inspiratory muscles, leading to increased oxygen costs of breathing. Although this shallow and rapid ventilatory pattern may bypass counteracting elastic forces, the relative dead space ventilation increases [2, 4, 8, 9]. All these issues contribute to reducing the ventilatory efficiency during exercise in these patients [4, 10].

Bariatric surgery, especially sleeve gastrectomy (SG), is an increasingly recommended treatment option for patients with morbid obesity, leading to a reduction in the prevalence and incidence of several obesity-related comorbidities [11, 12]. Regarding the respiratory system, surgical treatment of obesity was associated with a significant improvement in pulmonary function and the associated respiratory complications [13–16]. However, there is limited evidence on the effect of massive weight loss on ventilatory response during maximal exercise, while most studies have mainly focused on resting lung volumes and capacity [17–20]. Moreover, no data on the specific impact of SG on ventilation are currently available, particularly during incremental exercise testing.

To our knowledge, this is the first study aiming to investigate the impact of SG and the associated weight loss on ventilation at rest and during exercise, evaluating respiratory function, breathing pattern, ventilatory efficiency and the effects on exercise capacity, in a homogeneous population of severely obese patients.

## Methods

This observational cohort study evaluated 46 patients with severe obesity. All study participants followed a regionally approved clinical pathway established for patients who were listed for and underwent SG (Veneto Region, resolution n.55/CR August 4, 2015). This study analysed observational data of this diagnostic-therapeutic pathway of clinical assistance between June 2017 and January 2019. All consecutive patients who were considered suitable candidates for SG after medical, surgical and functional evaluations were included in this study. Specifically, candidates were patients with II or III class obesity who, following the treatment algorithm proposed by the Italian Society of Obesity, were eligible for bariatric surgery [21]. After obtaining written informed consent, functional evaluation was performed about 1 month before and 6 months after SG. These assessments were part of the routine clinical approach, including surgical risk stratification and subsequent follow-up with a cardiopulmonary exercise test (CPET).

Patients older than 70 years of age, with an ASA physical status class IV as well as those refusing the surgical intervention, were excluded. Further exclusion criteria were psychotropic substance abuse, cardiovascular and orthopaedic diseases, which contraindicated or impaired the exercise test, and failure to perform a maximal CPET.

### Spirometry

Standardized resting spirometry was executed in sitting position before performing the CPET. For each patient, repeated spirometric testing was performed, selecting the best attempt for data analysis. The acquired values for forced vital capacity (FVC), expiratory flow in the first second (FEV_1_), peak expiratory flow (PEF), and maximal expiratory flow at 25–50–75% of the FVC (MEF_25–50–75_) were compared with the predicted values for age, gender and BMI.

### CPET

Exercise capacity and ventilatory response were assessed during incremental, ECG-monitored, maximal CPET. Tests were performed on treadmill, according to the modified Bruce protocol, with an integrated initial 5-min constant speed interval (2.7 km/h). The subsequent incremental part of the exercise test was performed until patients’ exhaustion, reaching a Borg rating of perceived exertion ≥ 18/20.

Respiratory gas exchange and ventilation (VE) were analysed breath-by-breath, averaging 20-s intervals during rest and incremental exercise. The physiologic dead space ventilation at peak exercise (VDc/VT_max_) was calculated from the end tidal carbon dioxide partial pressure (PETCO_2_) according to the N.L. Jones modification of the Bohr equation and corrected for apparatus dead space *VDc/VE = (PaCO*_*2*_*c – PECO*_*2*_*)/PaCO*_*2*_ *– BF × VDs/VE*, where VDs = apparatus dead space and PaCO_2_c is estimated from PETCO_2_ (*PaCO*_*2*_*c = 5.5 + 0.90 × PETCO*_*2*_ − *0.0021 × VT*) [22]. Ventilatory equivalents for oxygen (VE/VO_2_) and carbon dioxide (VE/VCO_2_) were determined at the first (anaerobic threshold, AT) and second ventilatory threshold (respiratory compensation point, RCP), respectively. VE/VCO_2_ slope was calculated by linear regression from the start of the test, excluding initial hyperventilation, until the RCP. Peripheral oxygen saturation (SaO_2_) was determined throughout the CPET.

### Statistical Analysis

Data were elaborated with IBM-SPSS. Data are presented as mean, median and standard deviation. The Shapiro-Wilk test was used to check for normal distribution within the dependent variables. The comparisons from pre- to post-SG were performed using paired *t* tests for normal distributed variables; otherwise, the Wilcoxon-Mann-Whitney test was performed. A *p* value < 0.05 was considered statistically significant.

## Results

A total of 46 (11 M; 35 F) patients with morbid obesity (BMI 43.59 ± 5.30 kg/m^2^, weight 122.12 ± 19.47 kg) were included in this clinical trial, with a mean age of 44.17 ± 11.22 years. At baseline, six patients (13%) were medically treated with oral hypoglycaemics (two with metformin and liraglutide, four only with metformin), while 14 patients (30%) were medically treated for arterial hypertension taking ACE inhibitors, Angiotensin II receptor blockers, Calcium channel blockers, Thiazides or an association of these. Six patients (13%) were asthmatic and were taking a combination of ICS/LABA and salbutamol on demand. Furthermore, 14 (30%) and 11 (24%) patients were active and former smokers, respectively. Also, 29 patients (63%) affirmed to conduct a sedentary lifestyle. However, after SG patients lost 32.14 ± 8.99 kg or 26.35 ± 6.17% of body weight, leading to a mean BMI of 32.27 ± 4.84 kg/m^2^.

### Pulmonary Function at Rest

Spirometric flows and volumes were on average above the lower limit of normal at baseline. After SG, there was a significant increase, both in volumes and forced dynamic flows (FVC, FEV_1_, MEF_25_; all *p* ≤ 0.001), while the Tiffeneau index was not significantly affected (Table 1). Resting ventilation was found decreased after massive weight loss, showing a reduced TV (*p* < 0.001), without a significant impact on breathing frequency (BF; Table 2).

**Table 1:** Spirometric parameters pre- and post-sleeve gastrectomy

	Evaluation pre-SG		Evaluation post-SG		
*n* = 46	Mean	SD	Mean	SD	*p* value
FVC (%)	103.85	± 12.84	111.95	± 12.31	*p* < 0.001
FEV_1_ (%)	98.65	± 15.87	106.33	± 14.65	*p* < 0.001
PEF (%)	97.96	± 17.11	100.17	± 16.14	*p* = 0.319
MEF_75_ (%)	99.50	± 24.73	102.28	± 22.95	*p* = 0.228
MEF_50_ (%)	87.39	± 32.69	90.87	± 32.67	*p* = 0.193
MEF_25_ (%)	62.80	± 26.91	73.39	± 32.60	*p* < 0.001
FVC (l)*	3.71	± 0.80	3.99	± 0.92	*p* < 0.001
FEV_1_ (l/s)*	2.99	± 0.73	3.21	± 0.83	*p* < 0.001
FEV_1_%FVC*	80.21	± 6.53	80.48	± 8.32	*p* = 0.760
MEF_25_ (l/s)*	1.18	± 0.61	1.38	± 0.81	*p* = 0.003

Forced spirometric volumes and flows were compared pre- and post-sleeve gastrectomy (SG). *FVC* forced vital capacity, *FEV*_*1*_ expiratory flow in the first second, *PEF* peak expiratory flow, *MEF*_*25–50–75*_ maximal expiratory flow at 25–50–75% of the forced vital capacity, *FEV*_*1*_*% FVC* Tiffeneau index. “*” indicates parameters not normally distributed

**Table 2:** Ventilatory response to exercise pre- and post-sleeve gastrectomy

	Evaluation pre-SG		Evaluation post-SG		
*n* = 46	Mean	SD	Mean	SD	*p* value
VE_rest_ (l/min)	14.54	± 3.69	10.30	± 3.50	*p* < 0.001
VE_AT_ (l/min)	41.46	± 10.23	33.37	± 7.56	*p* < 0.001
VE_max_ (l/min)*	83.41	± 19.38	75.65	± 16.69	*p* < 0.001
TV_rest_ (ml)*	830.20	± 261.41	613.11	± 235.62	*p* < 0.001
TV_AT_ (ml)*	1674.13	± 543.73	1494.89	± 477.42	*p* < 0.001
TV_max_ (ml)*	2173.04	± 595.79	2168.39	± 613.46	*p* = 0.793
∆TV_rest to AT_ (ml)*	843.93	± 437.04	881.78	± 451.14	*p* = 0.906
∆TV_AT to Peak_ (ml)*	498.91	± 271.55	673.50	± 324.77	*p* < 0.001
BF_rest_ (1/min)*	18.48	± 5.44	17.63	± 4.96	*p* = 0.306
BF_AT_ (1/min)	26.00	± 6.70	23.37	± 5.17	*p* = 0.001
BF_max_ (1/min)*	41.74	± 9.00	37.59	± 6.98	*p* < 0.001
∆BF_rest to AT_(1/min)*	7.52	± 5.72	5.73	± 5.22	*p* = 0.028
∆BF_AT to Peak_(1/min)*	15.74	± 6.63	14.22	± 6.02	*p* = 0.136
BR_max_ (%)	18.41	± 16.22	30.24	± 16.22	*p* < 0.001
VDc/VT_max_ (%)*	12.09	± 6.23	9.63	± 6.69	*p* = 0.015
VE/VO_2 AT_*	22.57	± 3.09	22.62	± 2.56	*p* = 0.739
VE/VCO_2 RCP_*^#^	27.00	± 3.24	26.25	± 3.09	*p* = 0.058
VE/VCO_2 slope_*	27.04	± 3.94	25.47	± 3.32	*p* = 0.002
PetCO_2 rest_ (mmHg)	33.76	± 3.43	30.51	± 3.11	*p* < 0.001
PetCO_2 AT_ (mmHg)	39.95	± 4.28	39.53	± 3.70	*p* = 0.384
SaO_2 rest_ (%)*	99.33	± 0.70	99.26	± 0.68	*p* = 0.597
SaO_2 max_ (%)*	97.89	± 1.29	98.52	± 0.75	*p* = 0.001

Effects of massive weight loss after sleeve gastrectomy (SG) on ventilation at rest and during incremental cardiopulmonary exercise testing. *VE* ventilation, *TV* tidal volume, *BF* breathing frequency, *AT* anaerobic threshold, *∆ rest to AT* delta of the BF/TV from rest to the anaerobic threshold, *∆ AT to Peak* delta of the BF/TV from the anaerobic threshold to peak exercise, *BR*_*max*_ breathing reserve at peak exercise, *VDc/VT*_*max*_ physiologic dead space/tidal volume at peak exercise, *VE/VO*_*2*_ ventilatory equivalent for oxygen, *VE/VCO*_*2*_ ventilatory equivalent for carbon dioxide, *RCP* respiratory compensation point, *PetCO*_*2*_ partial pressure of end-tidal carbon dioxide, *SaO*_*2*_ peripheral oxygen saturation. “*” indicates parameters not normally distributed. “^#^” indicates a sample size of 43 subjects because 3 patients have not reached the RCP

### Pulmonary Function During Exercise

**Fig. 1:**
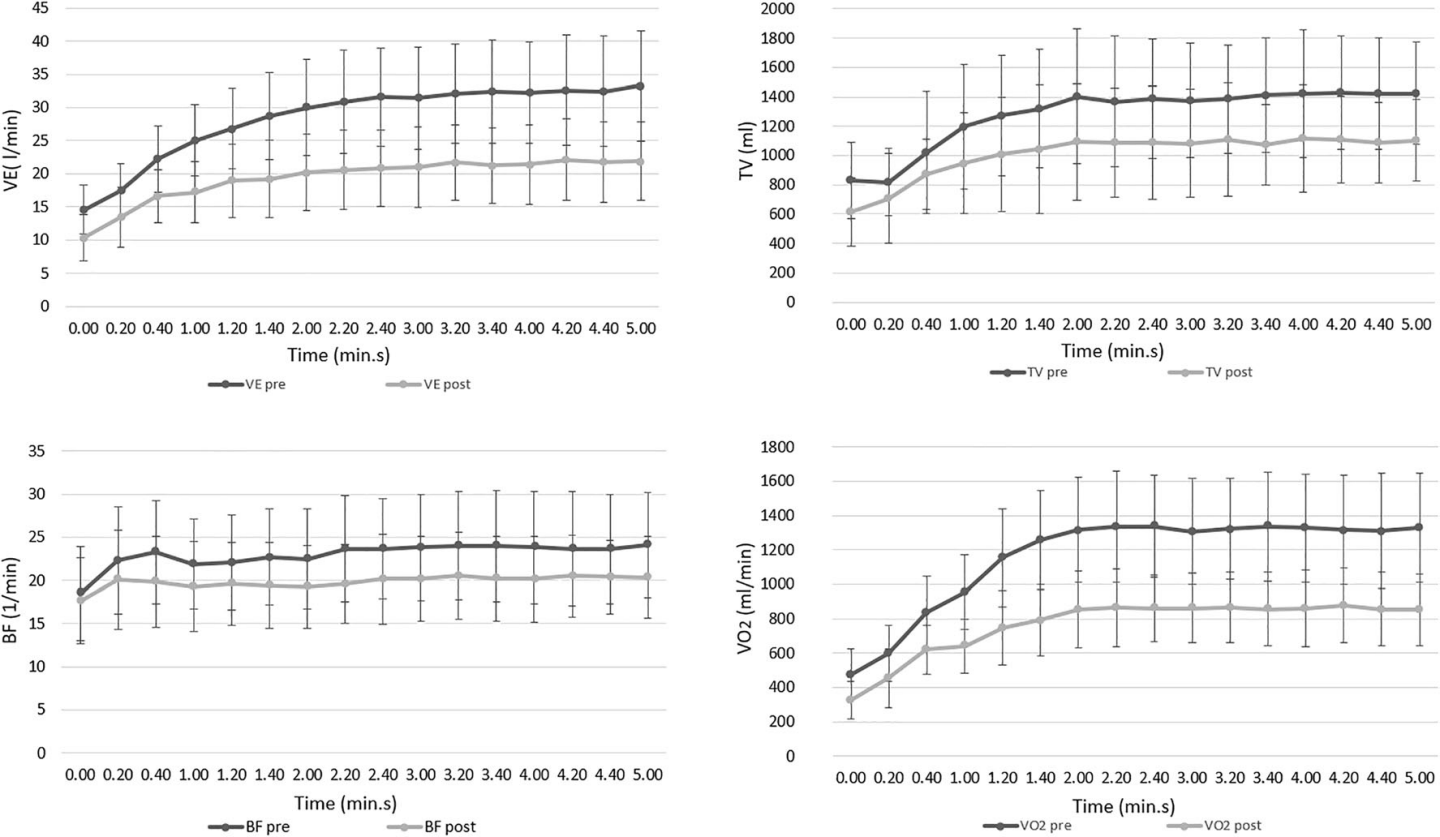
Ventilatory response and oxygen consumption at submaximal exercise. Ventilatory response and oxygen consumption during the initial 5 min of CPET at constant load, before and after sleeve gastrectomy (SG). Patients with severe obesity presented after massive weight loss a lower ventilatory response (VE), with reduced tidal volume (TV) and breathing frequency (BF). This was partially due to lower ventilatory demands because of decreased oxygen consumption (VO_2_). Data are presented as mean and SD

After SG, ventilation significantly decreased at all levels of incremental workload, which was associated, at submaximal exercise, to a reduced TV and BF (all *p* ≤ 0.001; Fig. 1, Table 2). However, the impact on patients’ ventilatory pattern is also shown by a lower increase of the BF at submaximal exercise (∆BF_rest to AT_ *p* = 0.028) and a larger response of the TV at vigorous/maximal exercise intensities (∆TV_AT to Peak_ *p* < 0.001). Analysing ventilatory efficiency, a concomitant improvement of the calculated dead space ventilation (VDc/VT_max_), VE/VCO_2_ slope and peripheral oxygen saturation was shown (all *p* < 0.015; Table 2).

These improved pulmonary flow/volume capacities and ventilatory efficiency were associated with a significantly increased breathing reserve at peak exercise (*p* < 0.001), also because the significant weight loss after SG decreased ventilation during exercise.

### Cardiorespiratory Fitness

The ventilatory modifications were associated with an increased exercise capacity and tolerance post-SG. Patients showed an improvement in exercise time and peak oxygen consumption per kilogram body weight (VO_2_/kg_peak_), as well as a higher peak respiratory exchange ratio (RER) (all *p* < 0.001; Table 3). However, a reduced oxygen demand was found after SG as shown by a statistically significant decrease in absolute VO_2_ at rest and during exercise (*p* < 0.001).

**Table 3:** Physical exercise capacity

	Evaluation pre-SG		Evaluation post-SG		
*n* = 46	Mean	SD	Mean	SD	*p* value
Exercise time (min)*	14.94	± 2.54	17.39	± 2.43	*p* < 0.001
VO_2 rest_ (ml/min)	476.15	± 148.99	326.54	± 111.64	*p* < 0.001
VO_2 AT_ (ml/min)*	1716.65	± 488.52	1353.19	± 317.10	*p* < 0.001
VO_2 peak_ (ml/min)*	2442.93	± 622.88	2205.15	± 565.92	*p* < 0.001
VO_2_/kg _rest_ (ml/min/kg)*	3.91	± 0.90	3.64	± 1.12	*p* = 0.227
VO_2_/kg _AT_ (ml/min/kg)	14.17	± 2.86	15.09	± 2.94	*p* = 0.040
VO_2_/kg_peak_ (ml/min/kg)*	20.27	± 3.84	24.70	± 5.67	*p* < 0.001
RER_rest_	0.81	± 0.06	0.76	± 0.08	*p* < 0.001
RER_max_*	1.12	± 0.09	1.18	± 0.09	*p* < 0.001

Effect of massive weight loss after sleeve gastrectomy (SG) on patients’ physical exercise capacity and tolerance. *VO*_*2*_ absolute oxygen consumption, *AT* anaerobic threshold, *VO*_*2*_*/kg* oxygen consumption per kilogram bodyweight, *RER* respiratory exchange ratio. “*” indicates parameters not normally distributed

## Discussion

### Pulmonary Function at Rest

The results of this clinical trial show that the massive weight loss after SG is associated with an improvement in mechanical restrictive ventilatory limitations. Previous studies attributed this obesity-related pulmonary impairment to excessive thoracic and visceral adipose tissue [8, 23, 24]. Although at baseline respiratory flows and volumes were substantially within normal limits, 6 months after SG, the unchanged Tiffeneau index in the presence of increased dynamic pulmonary parameters (FVC and FEV_1_) suggests a major impact of weight loss on restrictive components of ventilation. Analysing the pulmonary flows at different forced expiration levels and in accordance with the findings of Held et al., a more pronounced improvement was observed for small airways (MEF_25_), compared with the pulmonary flows in larger airways (MEF_50_ and MEF_75_) [25]. Rubstein et al. stated that obesity carries flow limitations that could be explained, among other mechanisms, by a state of systemic inflammation and an increase in pulmonary blood volume, which lead to congestion of vessels and a reduction in the size of small airways [4, 26]. Moreover, MEF_25_, which from a physiological point of view represents an objective measure of small airway resistance, is positively influenced by increased lung volumes [27]. Thus, an enhanced MEF_25_ could be due to an improvement of both the generalised inflammatory state and lung volumes, leading to a reduction of peripheral pulmonary resistances and of mechanical restrictive limitations.

Data also show a decrease in resting ventilation after SG that might reflect a reduction in global energy/oxygen demands and improved ventilatory efficiency. In fact, in accordance with previous studies, a reduction in resting absolute VO_2_ was found [28–30]. Furthermore, as also observed by Matos et al., the resting ventilatory pattern was characterized by a lower ventilation, mainly due to a reduction of the TV, which appears, however, sufficient to ensure adequate alveolar ventilation [19]. This suggests an optimization of ventilatory efficiency resulting from an improved pulmonary compliance and gas exchange [8]. Indeed, it is known that areas of atelectasis, frequently present in severe obesity, might be recovered and recruited for ventilation after massive weight loss [2, 4, 23].

### Pulmonary Function During Exercise

Regarding the ventilatory response to submaximal exercise, a few other studies have observed similar results after bariatric surgery, namely a reduction of ventilation for the same amount of external workload. Indeed, the ventilation during submaximal exercise post-SG was found reduced in both its components, i.e. BF and TV (Fig. 1). However, there is still a lack of evidence regarding the ventilatory response during incremental effort until exhaustion and the previously examined populations were different and, in part, not homogeneous [28, 29, 31, 32]. The shown significantly lower increase of BF (∆BF_rest to AT_) and the associated more pronounced response of TV (∆TV_AT to Peak_) after SG suggest a less shallow ventilatory response pattern during incremental exercise (Table 2, Fig. 1). These adaptations become advantageous as the relative calculated dead space ventilation (VDc/VT_max_) is probably decreasing not only as a result of the reduction in BF [33], but mainly because of the increased ability to exploit TV contributing to gas exchange. Indeed, alveolar ventilation can be improved more by increasing the breathing depth rather than by increasing the respiratory rate [34].

Patients’ ventilatory efficiency was subsequently also analysed by examining the ventilatory equivalents for oxygen (VE/VO_2_) and carbon dioxide (VE/VCO_2_). As also described by Wilms et al., there are no significant differences with regard to VE/VO_2_ at the anaerobic threshold after massive weight loss [35]. Nevertheless, the concomitant improvement of VE/VCO_2_ and VDc/VT_max_ might suggest that weight loss increased ventilatory efficiency, likely because of adaptations of ventilatory mechanics and gas exchange [36, 37]. Finally, the increase in peripheral oxygen saturation at peak exercise after SG, although clinically not significant, could further confirm an improvement in ventilatory efficiency [38].

The improved ventilatory pattern with the associated reduction of dead space ventilation and the likely better ventilatory efficiency will reduce ventilatory demand during exercise, which is also due to reduced oxygen costs/consumption. The patients’ potentially higher ventilatory capacity (i.e. maximum voluntary ventilation based on FEV_1_) along with the lower ventilatory volume during exercise lead to the shown increased breathing reserve at maximal effort after SG [33, 39]. Our findings suggest that ventilation is not a factor limiting exercise in these patients. However, after a massive weight loss, the reduced ventilation and improved ventilatory efficiency lead to a lower work of breathing. Indeed, patients who can rely on major breathing reserves are less constrained by ventilation [8].

### Cardiorespiratory Fitness

Results of the present and previous studies show that the substantial weight loss after SG led to improved exercise capacity and tolerance [17, 35, 40, 41]. However, despite the associated increase in peak VO_2_/kg, absolute VO_2_ decreased at all exercise intensities (Table 3; Fig. 1). This is probably explained by a decrease in energy/oxygen demands due to a lower weight-related workload, lower costs of breathing and by a quantitative loss in lean mass that generally accompanies the loss of adipose tissue [42]. Furthermore, the decrease in absolute VO_2_ could also be influenced by a qualitative alteration of skeletal muscle function, i.e. a deterioration of peripheral oxidative muscle metabolism [28, 30, 43, 44].

### Limitations and Perspectives

The findings of this observational study refer to the first 6 months after SG; long-term studies are therefore needed to investigate whether the effects on ventilation and aerobic capacity are long-lasting. Additionally, the impact of SG on ventilatory efficiency should be further investigated by specifically designed clinical trials, evaluating pulmonary perfusion, diffusion and ventilation. Finally, the effects of bariatric surgery on obesity-related ventilatory disorders, such as obesity-hypoventilation syndrome or obstructive sleep apnoea syndrome, should be addressed by future research.

## Conclusion

This is the first study to investigate the impact of SG on ventilation at rest and during incremental exercise, evaluating respiratory function, breathing pattern/efficiency and the effects on exercise capacity, in a highly selected and homogeneously treated study population of severely obese patients. It is possible to affirm that the massive weight loss during the first 6 months after SG leads to an improvement in respiratory restrictive limitations and in ventilation mechanics that contribute to a more efficient ventilation both at rest and during exercise. The subsequent lower ventilation can also be explained by a reduction in oxygen demand. Moreover, the decreased ventilatory response to exercise might be due to an increased ventilatory efficiency with a less shallow breathing pattern and a reduced oxygen consumption during incremental exercise intensities. The adaptations described lead to an increased breathing reserve at peak exercise, which might indicate from a clinical point of view that patients’ exercise capacity is less prone to be impaired by ventilation. Finally, even though exercise capacity and tolerance improved after SG, the significant decrease of absolute oxygen consumption may reflect the associated loss of functional muscle mass.
